# Over 95% of large-scale length uniformity in template-assisted electrodeposited nanowires by subzero-temperature electrodeposition

**DOI:** 10.1186/1556-276X-6-467

**Published:** 2011-07-23

**Authors:** Sangwoo Shin, Bo Hyun Kong, Beom Seok Kim, Kyung Min Kim, Hyung Koun Cho, Hyung Hee Cho

**Affiliations:** 1 Department of Mechanical Engineering, Yonsei University, Seoul, 120-749, Korea; 2 School of Advanced Materials Science and Engineering, Sungkyunkwan University, Suwon, Gyeonggi-do, 440-746, Korea

**Keywords:** Cu nanowire, template-assisted electrodeposition, length uniformity, low-temperature electrodeposition, nucleation, crystallinity

## Abstract

In this work, we report highly uniform growth of template-assisted electrodeposited copper nanowires on a large area by lowering the deposition temperature down to subzero centigrade. Even with highly disordered commercial porous anodic aluminum oxide template and conventional potentiostatic electrodeposition, length uniformity over 95% can be achieved when the deposition temperature is lowered down to -2.4°C. Decreased diffusion coefficient and ion concentration gradient due to the lowered deposition temperature effectively reduces ion diffusion rate, thereby favors uniform nanowire growth. Moreover, by varying the deposition temperature, we show that also the pore nucleation and the crystallinity can be controlled.

## Introduction

Length uniformity in template-assisted electrodeposited nanowires is of great importance in realizing the nanowires as building blocks for various technological applications that specifically involve electron flow through the nanowires such as thermoelectric [1,2], spintronics [3,4], photovoltaics [5], interconnects [6,7], and phase-change memory devices [8]. In feasibility aspect, template-assisted nanowires have an extraordinary advantage over other nanowires that are grown by different methods such as vapor-liquid-solid method [9] and electroless-etched method [10,11]. In detail, the template can be readily used as a supporting matrix without any further fabrication process. Supporting matrix, which is essential in fabricating nanowire-based electronic devices, provides mechanical robustness, electrical insulation among adjacent nanowires, and fabrication feasibility when contacting the electrodes at the ends of the nanowires [12].

Ideally, template-assisted electrodeposited nanowires should all be connected to the conducting electrodes at the both ends. At one end, the seed layer that works as a working electrode holds all nanowires, but the problem arises from the other end. Nonuniform ion transport that is near and inside the template pores results in nonuniform growth of the nanowires. This in turn creates only a few numbers of nanowires that are fully grown to be exposed out of the template. By continuing the electrodeposition to further grow the premature nanowires, the fully grown nanowires start to create semi-spherical caps that completely block the other remaining pores having shorter nanowires, especially in widely used porous anodic aluminum oxide (AAO) due to its high porosity and packing density. This is extremely critical in fabricating the nanowire-based devices since only a limited numbers of the nanowires are actually in contact at the both ends. In other words, major portion of the nanowires is malfunctioning.

Several attempts on the uniform growth of template-assisted electrodeposited nanowires have been made recently such as pulsed electrodeposition [13-15], low-temperature electrodeposition [15], ultrasonic milling [16], and applying forced convection to the bulk electrolyte [17]. The most successful result achieved so far to the authors' best knowledge is from Stacy and co-workers where they achieved up to 93% of length uniformity in Bi_2_Te_3 _nanowires with length of 62-68 μm by employing pulsed electrodeposition in a well-ordered homemade AAO template at low deposition temperature (1°C to 4°C) [15]. Among the above-mentioned methods used to enhance the length uniformity, lowering the deposition temperature is shown to be highly effective, easily manipulated, and straightforward to proceed [15]. However, intensive study regarding the temperature effect on the growth of template-assisted electrodeposited nanowires is rarely done despite its importance [16,18,19].

Herein, we report highly uniform growth of template-assisted electrodeposited copper (Cu) nanowires by lowering the deposition temperature down to subzero centigrade. Cu nanowire is regarded as potential candidates for various applications including interconnects [6,7], giant magnetoresistive materials [3,4], infrared polarizer [20], and cooling applications [21,22]. The ion diffusion rate, which directly influences the electrodeposition behavior thereby length uniformity, can be appropriately controlled by varying the deposition temperature. Moreover, we show that the pore nucleation and the crystallinity can also be controlled by varying the deposition temperature.

## Experimental section

Commercially available AAO, Anodisc™ (nominal pore size 20 nm, Whatman, Maidstone, Kent, UK), which is about 60-μm thick, was used as an electrodeposition template. Anodisc™ can additionally give the most harsh electrodeposition conditions since this template exhibits extremely disordered form where the pores are nonuniformly distributed with different pore sizes at the each pore ends (see Figure A1 in Additional file 1) [15]. Also, the pores are seriously interconnected and defects are often observed. The overall pore size is about 200 nm to approximately 300 nm and the pores are spanned along the whole template. However, at the one end, the pores are separated into several smaller pores having pore size of about 20 nm to approximately 40 nm. In sum, the use of Anodisc™ can provide a lower limit of nanowire uniformity in growth of large-area nanowires by electrodeposition.

Cu was chosen to exclude any complex reagents and additives that may influence the ionic transport behavior. The aqueous Cu electrolyte simply consisted of 220 g/l CuSO_4_·_2_O (99%) and 32 g/l H_2_SO_4 _(95%) with highly deionized water (18.2 MΩ cm). All chemicals were purchased from Duksan Pure Chemicals (Seoul, Korea) and used as received without further purification process.

A three-electrode electrochemical cell was employed for Cu electrodeposition. A 300-nm Au with 20 nm of Cr adhesion layer was coated on one side of the template (narrow pore end) using e-beam evaporator to act as a working electrode. Ag/AgCl (saturated KCl) and Pt mesh electrodes were employed for reference and counter electrodes, respectively. All potentials in this study are given in relation to the standard hydrogen electrode (SHE) since the electrode potential of the Ag/AgCl electrode is temperature dependent [23]. To calibrate the temperature effect, linear temperature coefficient of the Ag/AgCl electrode versus SHE is given as -1.01 mV/K [23]. Linearvoltametry and potentiostatic electrodeposition were done using a standard potentiostat (VersaSTAT3, Princeton Applied Research). The deposition temperature was controlled in the range of -2.4 to 60.5°C using a constant temperature circulating bath (HI-1030I, Hanil Industrial Machine, Seoul, Korea) and the temperature was precisely maintained under ± 0.1°C of deviation.

After electrodeposition, nanowire-embedded AAO template was cleaved and the cross section was observed using an optical microscope (BX51M, Olympus, Olympus America, Inc., Center Valley, PA, USA) to verify the large-area length uniformity of the nanowires. Subsequently, the Au/Cr working electrode was partially etched away and placed into the scanning electron microscope (SEM; S-4300, Hitachi Co., Tokyo, Japan) and the X-ray diffractometer (XRD; Rint-2000, Rigaku Corporation, Tokyo, Japan) in order to determine the pore nucleation and the preferential nanowire growth direction, respectively. For XRD measurement, Cu-Kα radiation source at 40 kV and 30 mA was used in 2*θ *range of 30° to approximately 90° with scan rate of 0.02°/s.

The crystallinity of the individual Cu nanowires was observed using transmission electron microscope (TEM; JEM-3010, JEOL, Tokyo, Japan). Individual nanowire samples were prepared by dissolving the AAO template in 1 M NaOH for 1 day followed by thorough rinsing with an absolute ethanol and 10 min of sonication.

## Results and discussion

**Figure 1:**
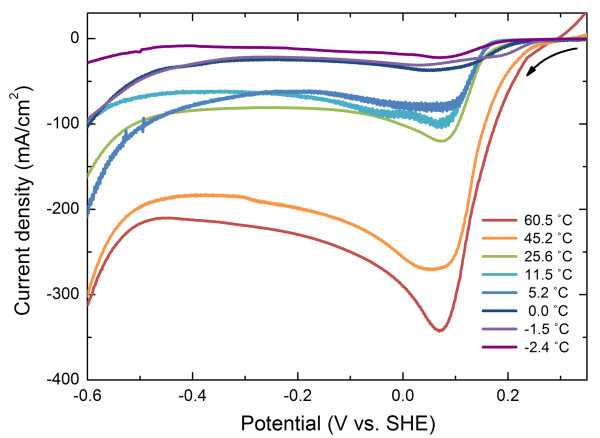
**Linear voltammograms of the Cu nanowires under varying temperature**. The scan rate is 0.1 V/s.

Prior to conduct potentiostatic electrodeposition, linearvoltametry was employed with scan rate of 0.1 V/s to investigate the appropriate reduction potential range of the Cu nanowires. Figure 1 shows the measured linear voltammogram. The results show that the temperature does not critically affect the Cu reduction potential which is in good agreement with previous study [24]. Reduction peak of Cu occurred in the range of 0.02 V to approximately 0.1 V. Therefore, we fixed the applied potential at 0.05 V and conducted potentiostatic electrodeposition throughout the whole temperature range.

**Figure 2:**
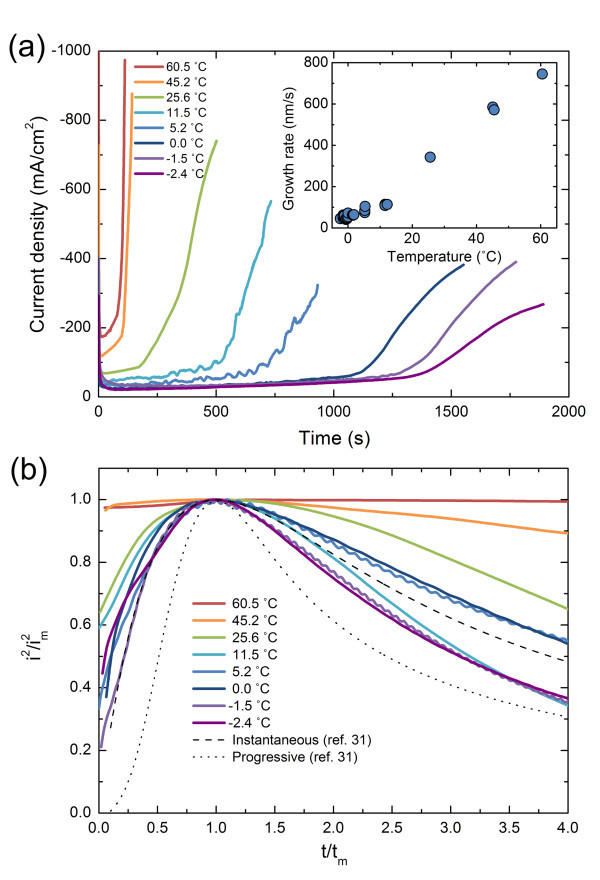
**Transient electrodeposition current density curves of the Cu nanowires under varying temperature**. (**a**) Full scale transient curves. Inset shows nanowire growth rate as a function of deposition temperature; (**b**) transient curves normalized by current and time maxima at the initial stage of the electrodeposition. Theoretical values for instantaneous and progressive nucleation models are also presented [31]. Dashed curve indicate instantaneous nucleation whereas dotted curve indicate progressive nucleation. *i_m _*and *t_m _*denote current maximum and its corresponding time.

Figure 2a shows electrodeposition current density transient curves for the Cu nanowires at various deposition temperatures. Briefly mentioning the electrodeposition stages are: (1) charging of electric double layer and subsequent development of diffusion layer at the vicinity of the surface of the working electrode which leads to an instantaneous rise and drop of the initial current density; (2) growth of nanowires inside the template where the current density remains steady; (3) overgrowth of the nanowires after reaching the pore end which leads to gradual increase of the current density due to the increase of the electrodepositing area. The nanowire growth rate can be derived from the time taken at the stage 2 which is presented in the inset. Inside the long and narrow pore channels, diffusion is the rate-determining process in electrochemical reactions [25]. The diffusion rate can be determined by the Fick's first law of diffusion where it is expressed as *j *= -*D*Δ*C *where *j *is the diffusion rate, *D *is the diffusion coefficient, and *C *is the local concentration. As the temperature is decreased, the diffusion coefficient of the Cu cations is also decreased since it follows the Arrhenius plot [26,27]. Moreover, not only the diffusion coefficient is decreased but also the thickness of the diffusion layer is elongated as the deposition temperature is decreased [28,29] since the nanoscale pore channels having aspect ratio up to 300:1 show a diffusion limited transport behavior [25]. In other words, concentration gradient at the diffusion layer is decreased. From the Fick's first law of diffusion, these two factors lead to the decrease of the mass transport rate. Therefore, by changing the deposition temperature, the nanowire growth rate can be significantly varied. At 60.5°C, it takes about 80 s to reach and fill the pore and the growth rate of the Cu nanowires is estimated as 745 nm/s whereas the growth rate is decreased down to about 45 nm/s at -2.4°C which is more than 16-fold decrease.

**Figure 3:**
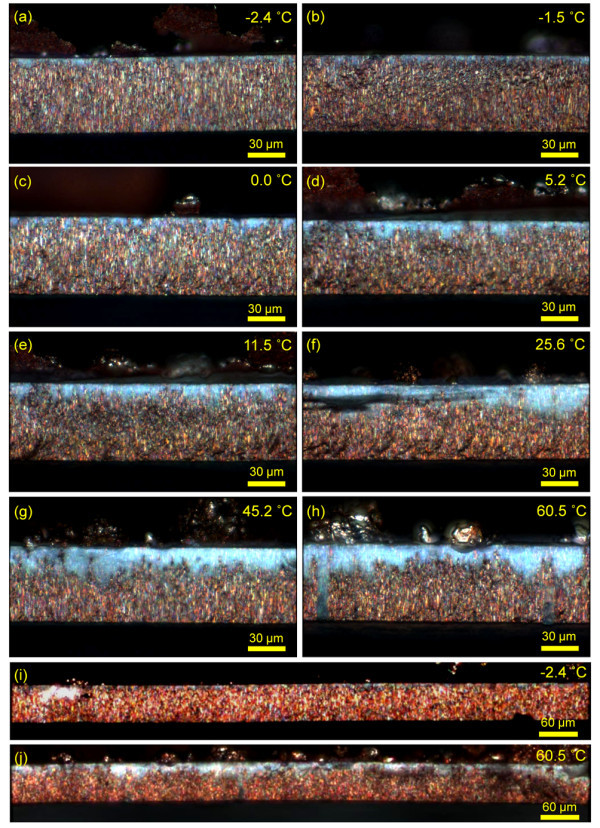
**Optical microscope images of the cross sections of the AAO template with Cu nanowires**. These are grown at various deposition temperatures: (**a**) -2.4°C; (**b**) -1.5°C; (**c**) 0.0°C; (**d**) 5.2°C; (**e**) 11.5°C; (**f**) 25.6°C; (**g**) 45.2°C; (**h**) 60.5°C. (**i**) and (**j**) show large-scale view of the cross sections at -2.4°C and 60.5°C, respectively. Yellow arrows indicate empty pores.

After electrodepositing the Cu nanowires at different temperatures, the cross sections of the nanowire-embedded AAO templates were observed to determine the large-scale length uniformity. The length uniformity was determined by obtaining the average nanowire length from the nanowire growth front and dividing by the length of the template. Nanowires were sufficiently deposited with enough time so that the Cu overdeposits were fully exposed on the top surface of the template. Figure 3 shows the optical microscope images of the nanowire-embedded AAO templates with varying deposition temperature. It is clearly seen that the overall length uniformity of the Cu nanowires are significantly enhanced as the deposition temperature is decreased. At high deposition temperature condition, the growth front was shown to be very disordered and fluctuating while highly uniform growth front which is located near the top edge of the AAO template was observed at subzero centigrade temperature conditions. At 60.5°C, only about 65% of the total length of the template is filled while more than 95% of the nanowire length uniformity is achieved at -2.4°C. Considering that these samples are potentiostatically electrodeposited and the AAO template used in this work is extremely disordered, this result is remarkable. Note once again that Stacy and co-workers recently achieved about 93% of nanowire length uniformity by pulsed electrodeposition in a well-ordered homemade AAO template when the deposition temperature was between 1°C and 4°C [15].

This extremely high length uniformity in a disordered AAO template is mainly attributed to the slow nanowire growth rate which is achieved by reducing the ion transport rate at low temperature. The slow ion diffusion may inhibit two major ion transport regions that can lead to the nonuniform nanowire growth: First is the transport of cations from the bulk electrolyte to the pore entrance. At room temperature, without any forced convection, cations entering the pores tend to be concentrated at the edge area of the template since the excess cations are constantly diffused from the adjacent bulk electrolyte due to the hemispherical diffusion layers formed at the edge area [17]. Second is the cation transport along the pore channels. Different pore diameters and native defects inside the pores can lead to nonuniform ion transport [15,17], resulting nonuniform nanowire growth. These two unfavorable transport behaviors are believed to be significantly hindered even without any stirring or pulsed electrodeposition when the deposition temperature is sufficiently lowered, thereby achieving extremely high length uniformity.

It is also known that the pore nucleation is dependent on the deposition temperature [17]. It is interesting to note that the Anodisc™ template may provide an effective way to evaluate the pore nucleation. As mentioned earlier, Anodisc™ exhibits interbranched pore structures at the narrow-end side of the template. As the electrodeposition proceeds, the fastest nucleated pore will reach the main (wide) pore and eventually block all the other narrow pores that share the same main pore. The narrow end is about 100 nm to approximately 200 nm long (Figure A1(f) in Additional file 1) and regarding the fast growth rate, this narrow end will be filled in a very short period of time, especially when the deposition temperature is high. By blocking other pores, we can easily distinguish the pores that are not nucleated at the initial stage since the cations cannot be supplied to the non-nucleated pores. Therefore, this narrow feature may provide an effective means of evaluating the instantaneous pore nucleation.

**Figure 4:**
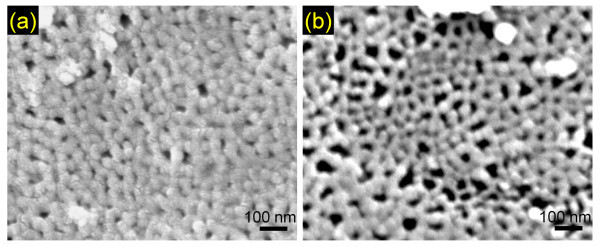
**SEM images of backside of the AAO templates with Au/Cr seed layers partially etched away**. Dark areas indicate empty pores. (**a**) -1.5°C; (b) 60.5°C.

To determine the pore nucleation, the back side of the nanowire-embedded AAO templates with Au/Cr layer partially etched away were observed using SEM which is presented in Figure 4. It can be clearly seen that at 60.5°C empty pores were easily observed whereas over 90% of the pores were nucleated at -1.5°C. Obviously, this is attributed to the different growth process of the Cu nanowires at various deposition temperatures. At high deposition temperature, the initial nucleation process is mainly instantaneous where the nuclei are mainly formed in the initial voltage pulse [30]. However, by lowering the deposition temperature, the initial nucleation process tends to shift toward progressive nucleation mode in which the nuclei are continuously formed during the nanowire growth. This can be verified by analyzing the current maxima at the initial stage of the electrodeposition curve in Figure 2b where two different nucleation modes are presented along with experimental data in a normalized form [31]. When progressively nucleating, the chances of pore nucleation during the nanowire growth are higher than instantaneous nucleation since the nuclei are continuously formed during the nanowire growth, even after the initial stage. Moreover, as mentioned earlier, slow nanowire growth also favors more pores to be nucleated before the pores are blocked. Therefore, by lowering the deposition temperature, a large-scale length uniformity and pore filling can be simultaneously achieved.

This different nucleation and growth processes can also affect the crystallinity of the nanowires [18]. In order to investigate the influence of deposition temperature on the crystallinity of the electrodeposited Cu nanowires, we conducted XRD and TEM studies. In general, it is well known that the electrodeposited nanowire exhibits a polycrystalline nature when grown potentiostatically [32,33]. However, the crystallinity can be enhanced to a certain degree by increasing the deposition temperature [18]. When the deposition temperature is low, slow growth rate promotes increased nucleation sites and growth of new grains. In contrary, high deposition temperature favors the growth of pre-existing grains which leads to the deposition of single crystals rather than the creation of new grains, thereby enhancing the crystallinity [18].

**Figure 5:**
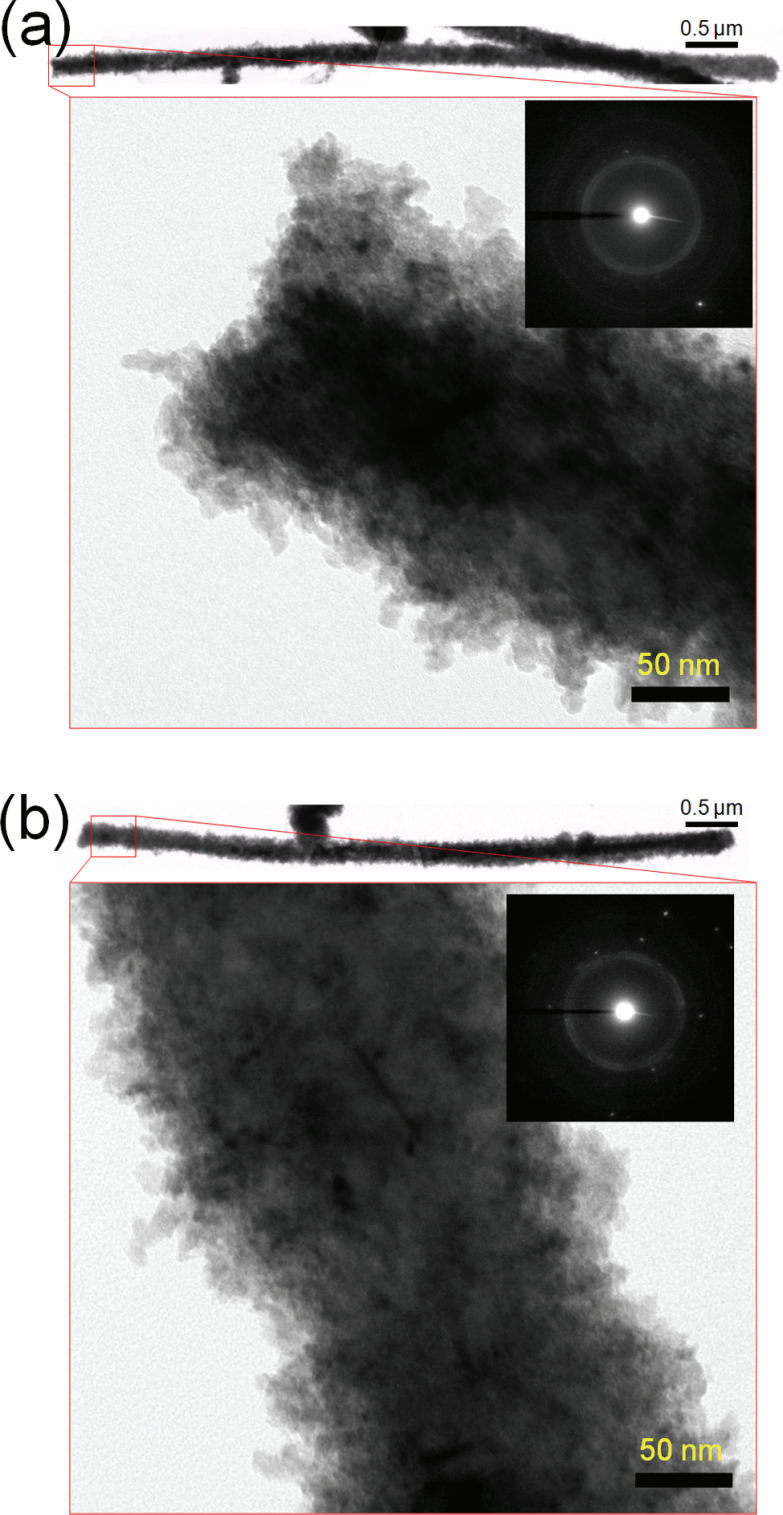
**TEM images of the Cu nanowires at various deposition temperatures**. (**a**) -1.5°C; (**b**) 60.5°C. Insets are the selected area electron diffraction patterns.

However, XRD results (Figure A2 in Additional file 1) showed that the overall grain orientation and the preferential growth direction were not significantly changed with deposition temperature. This is probably due to the fact that the electrodeposited nanowires are generally polycrystalline in nature, and XRD can only give the overall sum of multiple crystallographic orientations, which implies that without a significant enhancement of crystallinity (single crystalline), the diffraction patterns should not be varied much. The preferential growth direction of Cu nanowires was (111) throughout the whole temperature range. The TEM images of the Cu nanowires either from Figure 5a or 5b showed that a large number of spotty contrasts were observed with rough surface morphology regardless of the deposition temperature, which is typically observed in electrodeposited nanowires [33-36]. Obviously, this implies that the nanowires are in polycrystalline nature where the growth process is based on a typical 3D nucleation-coalescence mechanism [30,37]. However, the selected area electron diffraction patterns which are presented in the insets of Figure 5 showed an enhancement in the crystallinity with increasing deposition temperature where more enhanced diffraction spots were observed and the polycrystalline ring patterns were subsequently weakened. This directly indicates that the higher deposition temperature induces more favorable growth of existing grains that results in enhanced crystallinity, which is in conjuction with nucleation mechanism as mentioned earlier.

## Conclusion

In summary, we have achieved length uniformity of template-assisted electrodeposited nanowires over 95%, even in highly disordered commercial AAO template, when the deposition temperature was lowered down to subzero centigrade degrees. Due to the decreased ion diffusion rate and thereby decreased nanowire growth rate, uniform electrodeposition was enabled. Moreover, when the deposition temperature was lowered, pore nucleation was also significantly enhanced whereas the crystallinity was slightly decreased as the nucleation mechanism tended to proceed toward progressive nucleation. Therefore, by lowering the deposition temperature, large-scale length uniformity and pore filling can be simultaneously achieved which are both extremely important in realizing the nanowire array as practical large-scale applications.

## Abbreviations

AAO: anodic aluminum oxide; Cu: copper; SHE: standard hydrogen electrode; SEM: scanning electron microscope; XRD: X-ray diffractometer; TEM: transmission electron microscope.

## Competing interests

The authors declare that they have no competing interests.

## Authors' contributions

SS and HHC conceived of the study, and participated in its design and coordination. SS carried out the experiments on fabrication and SEM/XRD measurement of the nanowires. BHK and HKC carried out TEM experiments. SS drafted the manuscript. BSK, KKM, and HHC revised the manuscript. All authors read and approved the final manuscript.

## Supplementary Material

Additional file 1 **Additional information: Over 95% of large-scale length uniformity in template-assisted electrodeposited nanowires by subzero-temperature electrodeposition**. Click here for file
