# Supplementary material for: Over 95% of large-scale length uniformity in template-assisted electrodeposited nanowires by subzero-temperature electrodeposition
Source: Nanoscale Res Lett. 2011 Jul 23;6(1):467. doi: 10.1186/1556-276X-6-467 (PMC3211979; doi:10.1186/1556-276X-6-467)

Additional Information

Over 95% of large-scale length uniformity in template-assisted electrodeposited nanowires by subzero-temperature electrodeposition

Sangwoo Shin[1], Bo Hyun Kong[2], Beom Seok Kim[1],
Kyung Min Kim[1], Hyung Koun Cho[2] and Hyung Hee Cho[1*]

[1] Department of Mechanical Engineering, Yonsei University, Seoul, 120-749, Korea
[2] School of Advanced Materials Science and Engineering, Sungkyunkwan University, Suwon, Gyeonggi-do, 440-746, Korea

*Correspondence: hhcho@yonsei.ac.kr
Fax: +82-2-312-2159
Tel: +82-2-2123-2828

Figure A1. Scanning electron micrograph images of the commercial AAO template, Anodisc^{TM}. (a)-(c) Wide pore ends; (d)-(f) narrow pore ends; (g) sideview of the middle section of the AAO. Interconnected pores and defects are often observed.

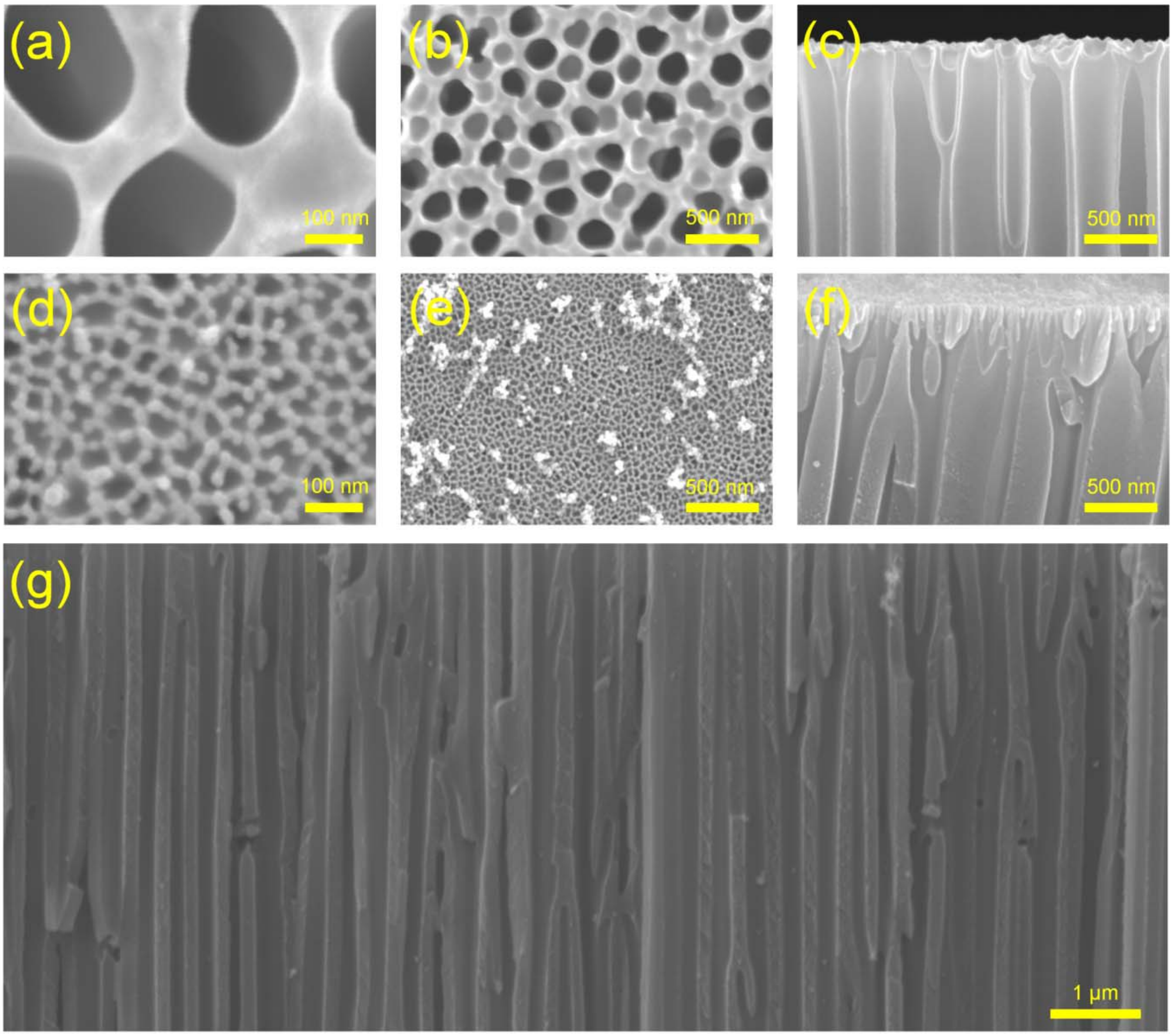

Figure A2. X-ray diffraction patterns of the Cu nanowires under various deposition temperatures. Red indices indicate Cu peaks (JCPDS 04-0836). Au (JCPDS 04-0784), Cr (JCPDS 06-0694), and CuI (JCPDS 01-0581) peaks are also observed. Au and Cr should be from incomplete etching. CuI is probably from the reaction of Cu with KI, which is an ingredient of the Au etchant.

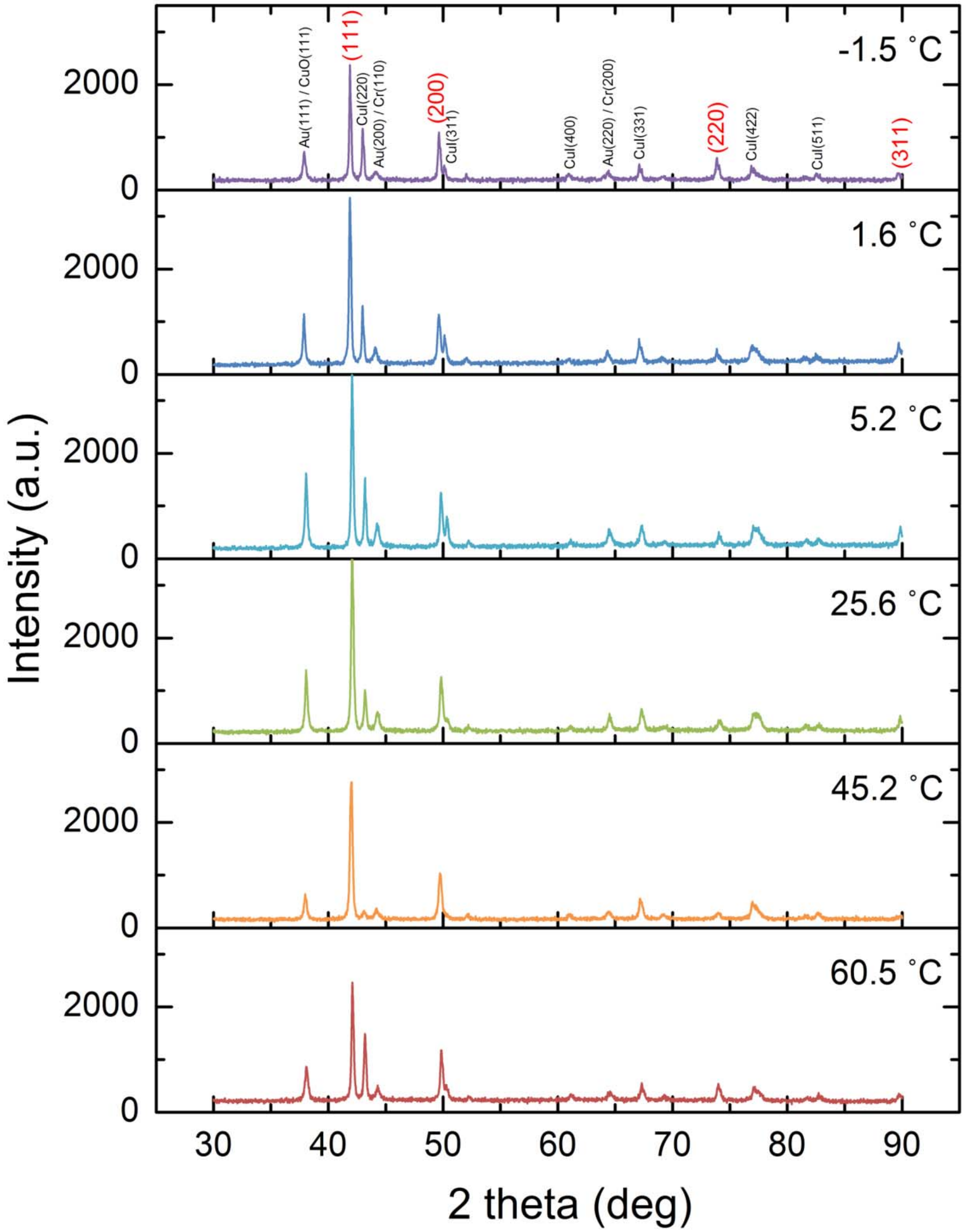

Supplement: Additional file 1 — Additional information: Over 95% of large-scale length uniformity in template-assisted electrodeposited nanowires by subzero-temperature electrodeposition. [file 1556-276X-6-467-S1.PDF]
